# Older adults’ refusal speech act in cognitive assessment: A multimodal pragmatic perspective

**DOI:** 10.3389/fpsyg.2023.1026638

**Published:** 2023-02-01

**Authors:** Lihe Huang, Huiyu Qu, Deyu Zhou

**Affiliations:** School of Foreign Languages, Research Center for Ageing, Language and Care, Tongji University, Shanghai, China

**Keywords:** refusal speech act, cognitive impairment, cognitive assessment, pragmatic compensation, examiner-patient interaction

## Abstract

This paper explores how older adults with different cognitive abilities perform the refusal speech act in the cognitive assessment in the setting of memory clinics. The refusal speech act and its corresponding illocutionary force produced by nine Chinese older adults in the Montreal Cognitive Assessment-Basic was annotated and analyzed from a multimodal perspective. Overall, regardless of the older adults’ cognitive ability, the most common discursive device to refuse is the demonstration of their inability to carry out or continue the cognitive task. Individuals with lower cognitive ability were found to perform the refusal illocutionary force (hereafter RIF) with higher frequency and degree. Additionally, under the pragmatic compensation mechanism, which is influenced by cognitive ability, multiple expression devices (including prosodic features and non-verbal acts) interact dynamically and synergistically to help older adults carry out the refusal behavior and to unfold older adults’ intentional state and emotion as well. The findings indicate that both the degree and the frequency of performing the refusal speech act in the cognitive assessment are related to the cognitive ability of older adults.

## 1. Introduction

With the burgeoning aging population in China, cognitive disorder with Alzheimer’s disease as the predominant type shows a high prevalence rate. Except for the related memory deficit studies, linguistic impairments have also received growing research interest at the early and prodromal states (Cuetos et al., 2007). Until recently, most linguistic studies have paid attention to the verbal communication between older adults with or without cognitive impairment and their caregivers or family members in clinical settings (Pilnick et al., 2018). However, few have focused on the examiner-patient communication in the cognitive assessment. The patient’s verbal and non-verbal communication with the examiner can reflect a wealth of information, such as cognitive ability, language ability, and social experience; it will to some extent impact the progressivity, accuracy, and results of the assessment. Therefore, it is worth noticing the academic and clinical significance of the examiner-patient interaction in the cognitive assessment and conducting further exploration into this.

This study focuses on the refusal speech act of Chinese older adults with different cognitive abilities in the cognitive assessment from the perspective of multimodal pragmatics. The study takes the RIF of older adults as the research object and applies a mixed approach to examine the features and influencing factors of different degrees of RIF. Multiple means of expression used by older adults, including non-verbal acts and prosodic features, to perform the refusal speech act will also be sketched. The study first divided the RIF into four levels regarding the degree of refusal behavior (Table 1) and applied a quantitative approach to explore the average frequency distribution of each level of RIF performed by older adults in every single turn during the examiner-patient interaction. Then, two expression devices (non-verbal acts and prosodic features) and two affecting elements (intentional state and emotional state) related to the performance of the refusal speech act were, respectively, examined through both quantitative and qualitative analysis. Last, the study proposed a preliminary interactive mechanism model of older adults performing the refusal speech act in the cognitive assessment from cognitive, linguistic, and psychological perspectives.

**Table 1:** Four levels of the RIF.

Level	Remark	Example
I	The speech content shows refusal, but the content itself is not relevant to any subtask or the whole task of MoCA-B.	*“I cannot see it clearly.”* (The speaker is asked to say the name “tiger” in the naming task.)
II	The speech content shows reluctance to perform the task with a task-related reason or excuse.	*“There is a lot of furniture.”* (The speaker is asked to recall the word “sofa” which belongs to the furniture category in the delayed recall task.)
III	The speech content reflects an inability or difficulty to perform or continue the task.	*“I cannot figure this out.”* (The speaker is asked to provide three combinations to pay 13 yuan in the calculation task.)
IV	The speech content reflects a kind of unwillingness to perform the task.	*“I do not want to see this.”* (The speaker is asked to identify objects and name them in the visuoperception task.)

## 2. Refusal speech act in clinical settings: A review

Communication in medical discourse is the basis for reaching a diagnosis, planning treatment, and giving advice (de Haes and Bensing, 2009). In the field of linguistics, researchers have revealed their interest in the study of doctor-patient communication (Menz, 2011), showing that effective doctor-patient communication is associated with positive outcomes, such as enhanced patient satisfaction, better treatment compliance, and better symptom resolution (Levinson et al., 2010; Liu et al., 2015). Applying linguistic theories and methodologies such as conversational analysis, cooperative principle, and multimodal approach to analyze doctor-patient conversations can effectively locate obscure speech structures and characteristics that may reflect communicative problems, and can help provide corresponding improvement measures to achieve effective doctor-patient interaction.

The patient’s refusal behavior in clinical settings has been conceptualized as a form of non-compliance that may present itself in specific acts such as questioning the clinician’s decisions, refusing treatment recommendations, or not providing relevant answers, which eventually obstruct the therapeutic work (Muntigl, 2013; Zhao and Ma, 2020). What enables refusal behavior to attract particular attention from researchers are its potential adverse effects, such as halting or undermining the progress of treatment or counseling, or hindering the success of doctors or counselors in achieving their aims, resulting in a poor doctor-patient relationship (Westra et al., 2012; Mamedova et al., 2020).

Some previous studies have focused on the various factors predicating for the patient’s refusal behavior in clinical settings. For instance, failure to communicate information to the patient, especially information such as the fact and purpose of the treatment, was found to easily elicit the patient’s refusal behavior (Appelbaum and Roth, 1983). The patient also refuses to maintain essential values such as self-esteem, affiliation, freedom, and health (Herrera et al., 2017). Sociological factors, including old age, low educational status, and nonprivate insurance, can influence the patient’s refusal behavior (Suh et al., 2017; Amini et al., 2020). Refusal management and corresponding communication strategies are also widely studied. Language strategies comprising making allusions to choices, using qualified propositions, and using plain language have proved effective in avoiding the patient’s resistance (Quick and Stephenson, 2008; Rains, 2013; Miller, 2015). Besides, Zhang (2021) summarized four discursive strategies to address the patient’s disalignment in online medical consultations in China: popularizing the mechanics of illness, providing reassurance, manifesting empathy, and delegating the responsibility of re-diagnosis.

In addition, many linguistic studies have delved into the patient’s refusal behavior in various medical agendas. However, in contrast to the high priority given to treatment and other medical agendas, the interaction between the examiner and the patient during the assessment process has received little attention (Elsey et al., 2015). What is lacking from the linguistic perspective is how refusal behavior becomes interactively displayed by older adults in the cognitive assessment, as well as the reasons behind it. Therefore, it is urgent to track the interactive progression of the cognitive assessment and figure out the characteristics, mechanisms, and causes of the refusal behavior of older adults.

Interpretations of the term “refusal” and the refusal speech act differ between scholars. This study follows (Chen et al.’s 1995) definition of “refusal,” i.e., refusal refers to a responding act in which the speaker resists participating in an action proposed by the interlocutor. On the one hand, the definition given by Chen et al. comprehensively covers the situations where a refusal speech act may occur. On the other hand, the refusal speech act is rarely found “alone,” but is accompanied by an “initial” utterance. Hence the refusal behavior can be seen as a “response” to the previous utterance, whether it is expressed verbally or non-verbally. In the clinical context of the cognitive assessment in this study, those speech acts that express a kind of resistance to engage in any subtasks or the entire assessment, or that hinder the proper advancement of the assessment, are considered the refusal speech act in question.

For different degrees of the refusal speech act, which may affect the listener’s subsequent response in interpersonal communication, most studies use a dichotomous approach, dividing refusals into strong and gentle ones. This classification ignores the multiple influencing factors and the complicated connotations when the speaker performs the refusal speech act. Besides, there are yet no consentaneous or sufficiently objective criteria for classification. The current study aimed to divide the RIF into four levels in terms of the degree of refusal behavior. The judgment of different levels was mainly based on the speech content of the RIF, while prosodic features and non-verbal acts were also considered as indicators strengthening or weakening the degree of refusal. Detailed remarks on the four-level categorization are found in Table 1.

Based on the above, the study takes the RIF performed by older adults during the assessment as the research object. Applying both qualitative and quantitative analysis, the study attempts to compare the characteristics of older adults with different cognitive abilities when performing the refusal speech act in the cognitive assessment from a multimodal perspective, and tries to answer the following questions:

1. Are there any differences in the degree and frequency of the RIF performed by older adults with different cognitive levels? If so, in what ways?
2. Are there any differences in the use of expression devices by older adults with different cognitive levels when performing the RIF? If so, in what ways?
3. What is the overall mechanism of older adults performing the refusal speech act in the cognitive assessment?

## 3. The present study: A multimodal pragmatics perspective

### 3.1. Data

The study takes the RIF as its research object, and the research data consists of 109 RIFs produced by nine Chinese older adults in the cognitive assessment of the Chinese version of Montreal Cognitive Assessment-Basic (MoCA-B). The data consists of about 122 min of audio and video data and around 30,500 words of transcribed texts.

All nine older adults’ cognitive abilities were confirmed by the authors’ university’s affiliated hospital, which categorized older adults into groups of normal control (NC, i.e., cognitively healthy older adults), mild cognitive impairment (MCI), and Alzheimer’s disease (AD), with each group consisting of three older adults. The nine older adults were 70.1 years old on average and were all female. The examiners in the cognitive assessment had all undergone the cognitive assessment training and examination, and had obtained professional qualifications approved by the Department of Neurology of the authors’ university’s affiliated hospital.

### 3.2. Material

The cognitive assessment material in the present study is the Chinese version of MoCA-B, translated and modified into a Chinese version by Chinese physician Guo Qihao (Guo and Chen, 2019). It was proposed to facilitate the application of the original MoCA among people with low education level or illiteracy (Julayanont et al., 2015). MoCA-B (Chinese version) can be downloaded from the official website.^1^ MoCA-B (Chinese version) has proven to be highly sensitive for the diagnosis of cognitive impairments in the Chinese context (Chen et al., 2016; Guo and Hong, 2016). The assessment evaluates eight cognitive domains: memory, language, attention, executive function, visuoperception, orientation, calculation, and abstraction, and usually lasts approximately 10–15 min (Guo and Hong, 2016). In this study, formally trained and skilled examiners administered MoCA-B (Chinese version) to older adults in a quiet environment. The examiners strictly followed the uniform instructions and steps of MoCA-B (Chinese version) without any differentiated guidance.

### 3.3. Methods

The analytical unit of this study is a single RIF, which is derived from the corresponding illocutionary act of refusal of speakers. When it comes to data segmentation and annotation, we use the term “performance unit” (Gu, 2013) to refer to the performance of each illocutionary act. In other words, an instance of a refusal speech act is equivalent to a performance unit in this study. The former is the object of study, which contains all the information and properties that a live refusal speech act holds, while the latter is a token that performs the refusal illocutionary act in live speech.

Operationally, the study first used Praat and Elan to segment and annotate data, and examined data validity and reliability by inviting two experts to evaluate the annotated data. Next, the study used the quantitative analysis to explore the differences in the frequency and degree of the RIF performed by older adults with different cognitive abilities. A combination of quantitative and qualitative approaches was then applied to analyze expression devices and affecting elements of the performance of the RIF. Last, the study aimed to propose an interactive mechanism to portray the interaction of various multimodal resources as well as the interplay of pragmatic ability, cognitive ability, and other aspects when older adults perform the refusal speech act in the cognitive assessment.

When it comes to data segmentation and annotation, the study applies Huang’s (2022) working scheme for multimodal pragmatic analysis of live illocutionary force. It is a scheme adopting the technology of multimodal corpus linguistics and the basic tenet of Simulative Modeling to emphasize the interaction between illocutionary force and prosody, emotion, non-verbal act, etc. In contrast to the previous research schemes of speech act that focus on the internal structure of language such as vocabulary and syntax, Huang’s scheme follows the idea that the interpersonal communication involves a multimodal interaction with the “live, whole person.” Moreover, the scheme breaks the ceiling of traditional speech act research, and provides a dynamic and multidimensional examination of illocutionary force.

**Figure 1:**
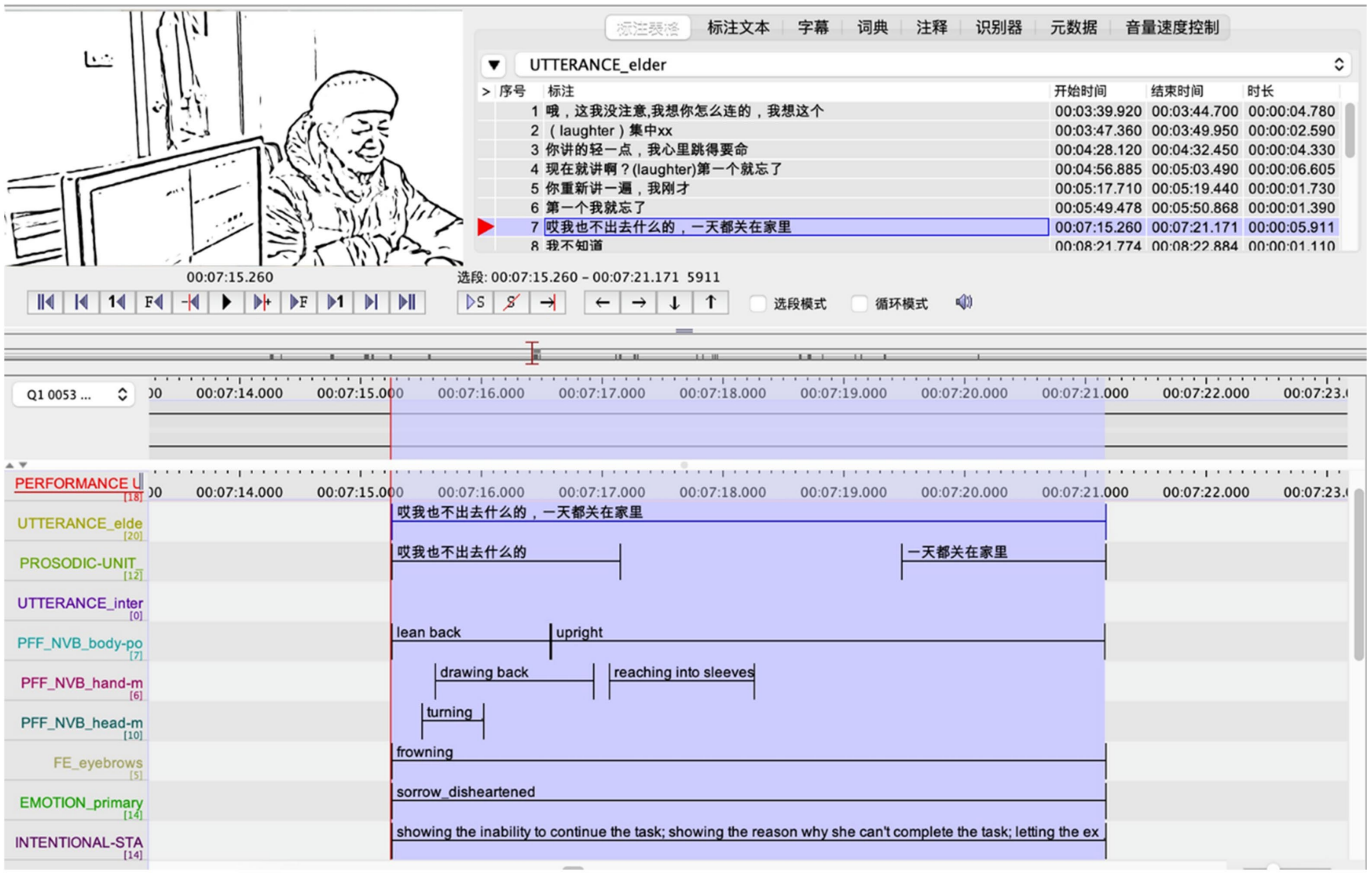
An annotated instance of the RIF, ELAN (Version 6.4) [Computer software] (2022).

In Huang’s (2022) scheme, thirteen tiers are established to annotate each illocutionary force. The tiers are: performance unit of illocutionary force, activity type, turn-taking, background emotion, primary emotion, social emotion, intonation group, prosodic pattern, other prosodic features, tasking-performance, non-verbal acts, intentional state, and interdependency. The working definition of each property is shown in Table 2. Four properties conforming to the target of the present study are selected as the annotation and analysis tiers in Elan, namely primary emotion, intentional states, prosodic features, and non-verbal acts. The specific labeling method of the four properties is shown in Table 3, and an annotated instance of the RIF is seen in Figure 1. The identification of both primary emotion and intentional states is based on various clues, including the speaker’s corresponding utterance content, prosodic features (e.g., intonational changes), and body language (e.g., facial expressions, gestures, postures). In terms of prosodic features and non-verbal acts, only those with obvious communicative function or directly related to the refusal behavior are examined. For example, the speaker may rub their hands together to keep warm, and this hand movement will not be counted and analyzed in the study; however, when the speaker rubs their hands out of nervousness or shyness, this hand-rubbing becomes an outward manifestation of emotion and hence should be examined. Besides, the present study places more emphasis on the presence or absence of a particular act or prosodic feature than on its duration.

**Table 2:** Thirteen tiers in the annotation scheme (Huang, 2022, pp. 112–141).

Property	Working definition
Performance unit of illocutionary force	A specific instance of a certain speech act type performed by a speaker in the specific situation.
Activity type	Any culturally recognized activity, whether that activity is coextensive with a period of speech or, indeed, whether any talk takes place in it at all (e.g., teaching, job interview, dinner party).
Turn-taking	Every continuous stream of speech given by each speaker in the situated discourse.
Background emotion	The emotion that hinges on the speaker’s physical condition and directly impacts the prosody. For example, a healthy speaker speaks loudly in a higher pitch on a specific occasion, indicating that the speaker is full of energy, while a speaker in poor health tends to speak in a low tone of voice, which sounds listless.
Primary emotion	As a universal subset of emotions, primary emotion spans cultures and races, occupying a central position, among other emotions. This study analyzed the seven most fundamental emotions: happiness, sadness, fear, anger, surprise, disgust, and worry.
Social emotion	The emotion that is closely related to the setting where the situated discourse is located and directly impacts illocutionary forces. Social emotions fall into three groups: positive, negative, and neutral, and there are two subsets in each group, i.e., other-directive and self-directive, which are to analyze whether a social emotion serves oneself or is stimulated by others.
Prosodic unit	The basic unit for analyzing and segmenting the performance unit, whose boundaries are segmented according to both the phonetic and grammatical or semantic cues.
Intonation patterns	The pitch, length, and loudness of the sound
Other prosodic features	The information of stress, pause, sound quality, and other para-linguistic cues, including laughter, sigh, cough, weeping, etc.
Task-doing acts	The acts taken by the speakers when they are performing a specific task, such as smoking, writing, pitching a tent, etc.
Non-verbal acts	The gestural information with intimate interaction with other modalities, such as gestures closely related to emotions and prosody (e.g., rocking backward while laughing). These non-verbal acts fall into four groups with a physical aspect: head movement, facial expressions, hand movement, and body movement.
Intentional states	Utterance is based on speaker’s intentions. The intentional states speakers might experience include intending a particular outcome, intending understanding from the hearers, intending understanding from the hearers entirely or partly resulting in their performance of perlocutionary acts, and attitudes to, beliefs about, hopes regarding, or requirements from others.
Interdependency	Consists of three aspects, including forward-and-backward interdependency (the relation between the speech act and the former/latter utterance), illocution-and-reality interdependency (the relation between the speech act and what is happening in the here-and-now behavior setting/beyond the here-and-now/both), and doing-and-talking interdependency (the relation between doing and talking).

**Table 3:** The labeling method of four properties.

Attribute name	Tag type	Tag value
Emotional state	Primary emotion	Anger
		Fear
		Sadness
		Disgust
		Surprise
		Happiness
		Worry
		Default
Intentional states	Primary intention	Believe{…}
	Attitude	Think{…}
	Belief	Want{…}
	Hope	Doubt{…}
Non-verbal acts	Head movement, “head”	Including nodding, shaking, and turning one’s head
	Facial expression, “expression”	Including smiling, crying, frowning, staring, mouth opening, and looking at/away
	Hand movement, “hand”	Including palm gesture (e.g., palms up), finger gesture (e.g., pointing), and shoulder gesture (shrugging)
	Body movement, “posture”	Including the movement of the stomach, back, legs, and the whole body
Prosodic features	Phonetic message	Stress
	Paralinguistic information	Laughter
		Coughing
		Clearing one’s throat

### 3.4. Data reliability and validity

After the segmentation and annotation, we exported the annotated data into a Microsoft Excel table. The table records a wealth of information including the speaker’s cognitive ability, the task type in which the RIF arises and its corresponding assessed function, and the annotated value. Then, two experts^2^ were invited to evaluate the reliability of the annotated data. The evaluation standard adopted the five-point Likert-type scales: one point for “strongly disagree,” two for “disagree,” three for “neither agree nor disagree,” four for “agree,” and five for “strongly agree.” The Wilcoxon signed-rank test was applied to examine the experts’ scores. The result showed that *p* = 0.123 (>0.05), indicating that there is no difference between the annotation done by the annotator and the two experts.

The data validity was evaluated after 109 instances of RIF found in the present study were categorized into different levels according to the remarks of four levels of the RIF in Table 1. Two experts separately scored each RIF based on the four-level categorization to evaluate whether the sub-levels are qualified in differentiating the degrees of the RIF. Cronbach’s alpha was applied to test the categorization’s validity, and the result turned out to be 0.997, which proved a high degree of consistency in the levels given by the two experts. This means that the four-level categorization has a high validity in reflecting the different degrees of the RIF.^3^

After the reliability and validity examination, the study further analyzed the data in terms of other prosodic features, non-verbal acts, intentional state, and primary emotion, and made a contrast among older adults with different cognitive abilities. The Mann–Whitney test and Kruskal-Wallis test were used to explore whether there were differences in the distribution of the RIF among older adults with different levels of cognitive ability.

## 4. Results

### 4.1. Frequency distribution of different levels of RIF

**Figure 2:**
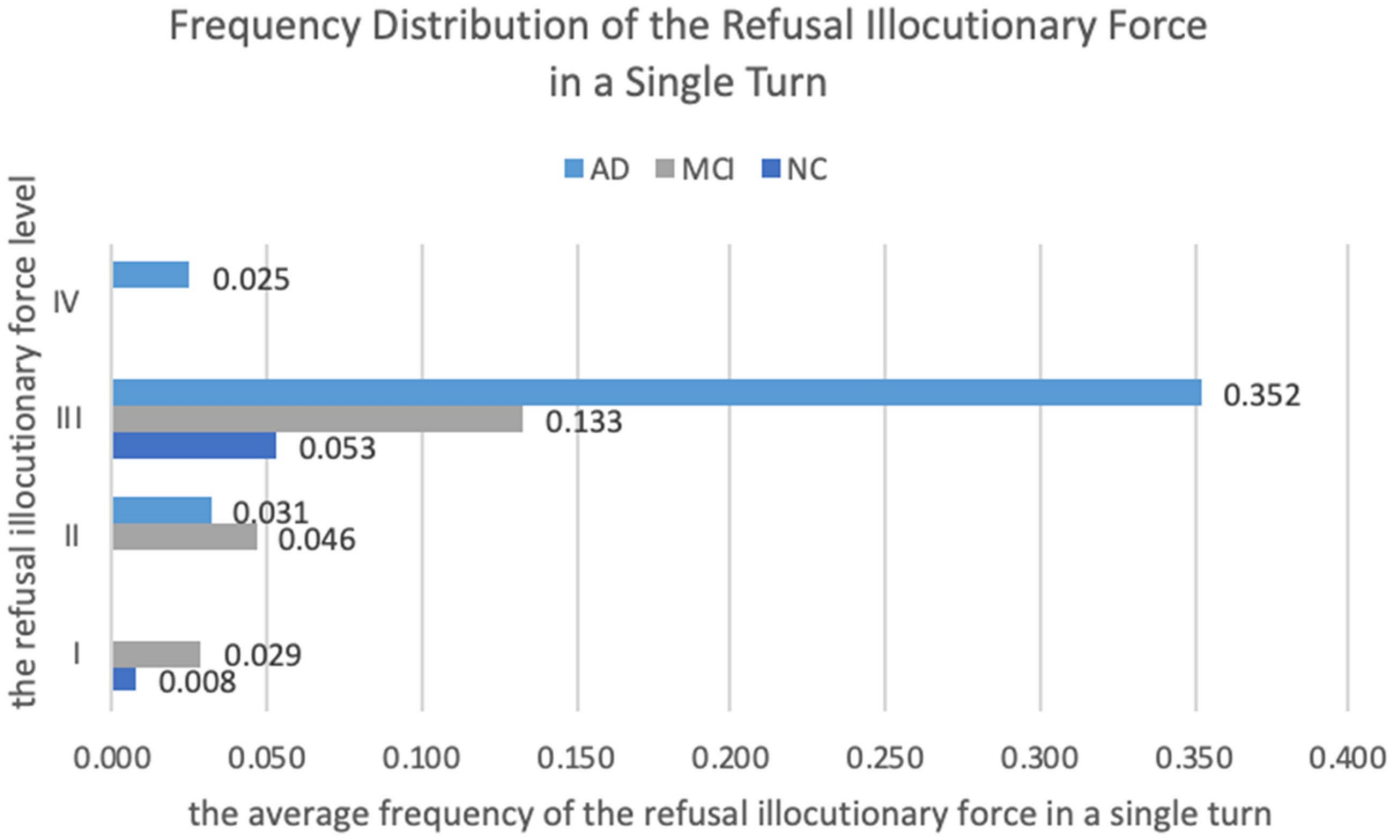
Frequency distribution of the RIF in a single turn.

To reduce the effect of the fact that older adults with different cognitive abilities tend to produce different amounts of discourse, this study calculated and compared the average frequency of the RIF in a single turn among the groups of NC, MCI, and AD to examine the differences. Generally, the switch of speaker is taken to define the timing of turn-taking. In other words, in this study, a single turn starts and ends with the same older adult. The frequency distribution of different levels of refusal behavior among the three groups is shown in Figure 2.

Descriptive statistics in Figure 2 reveal that the frequency distribution of the RIF in each level varied among different groups. The Mann–Whitney test and Kruskal-Wallis test were used to examine whether the differences were statistically significant, and the results are shown in Table 4. Since Level IV of RIF was only performed by the AD group (Figure 2), there was no non-parametric test for this level. Besides, the pairwise comparison was applied to Level III to examine the difference between every two groups, as shown in Table 5.

**Table 4:** Non-parametric tests.

Test	Level	Sig.
Mann–Whitney test	I	0.046*
	II	0.077
Kruskal-Wallis test	III	0.027*

*means statistically significant.

**Table 5:** Pairwise comparison.

Group	Sig.
NC-MCI	0.539
NC-AD	0.022*
MCI-AD	0.539

*means statistically significant.

As Figure 2 shows, Level I of RIF, whose speech content shows refusal but is not relevant to any subtask or the whole assessment, was present in both the NC and MCI groups, but abscent in the AD group. According to Table 4, the significance value between the NC and MCI groups was 0.046 (*p* < 0.05), indicating that there was a significant difference in the distribution of Level I between these two groups, with the MCI group performing Level I of RIF significantly more often than the NC group in a single turn. Similarly, as Figure 2 depicts, both the MCI and AD groups performed Level II of RIF (the speech content of the illocutionary force shows the reluctance to perform the task with a task-related reason or excuse), but the NC group did not. However, the significance value between the MCI and AD groups was 0.077 (*p* > 0.05; Table 4), indicating that although the MCI group performed Level II of RIF more frequently than the AD group, the difference was not statistically significant.

For Level III, i.e., the RIF whose speech content reflects an inability or difficulty to perform or continue the task, all groups frequently performed this level of RIF, and there was a significant difference in the distribution among the three groups (*p* = 0.027, <0.05, see Table 4). According to the following pairwise comparison (Table 5), this significant difference was located between the NC and AD groups, while the difference between the MCI group and the other two groups was not significant. That is to say, the AD group performed Level III of RIF significantly more often than the NC group. Lastly, Level IV of RIF, of which the speech content reflects a kind of unwillingness to perform the task, was only present in the AD group.

To sum up, both the degree and the frequency of the RIF were highest in the AD group, followed by the MCI group, and lowest in the NC group. The frequency of each level of RIF also varied among the three groups: (1) for Level I and Level II of RIF, both were more frequently performed by the MCI group than the others, (2) Level III of RIF was performed by all three groups, with the AD group performing it most frequently, and (3) only the AD group performed Level IV of RIF.

### 4.2. Expression devices of RIF

#### 4.2.1. Prosodic features

**Figure 3:**
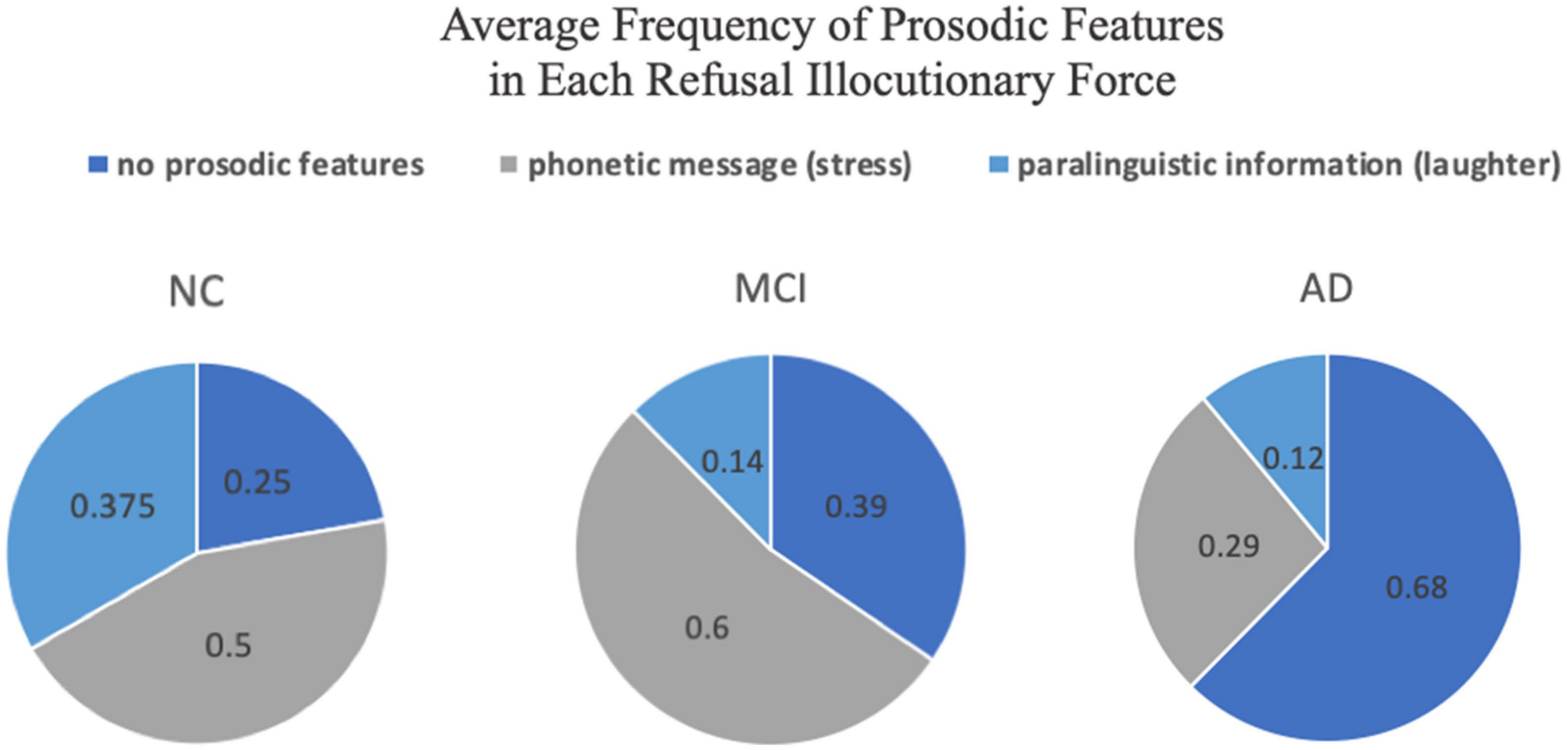
Average frequency of different types of prosodic features in each RIF.

The study focused on the phonetic message of stress and paralinguistic features, including laughter and coughing in data annotation, and calculated the average frequency of the found prosodic features in each RIF (Figure 3). The results show that when performing each RIF, both the NC group and the MCI group used stress most frequently, while no prosodic features were found in most cases of the AD group. In addition, only one prosody-related paralinguistic feature, laughter, was found in the data, which was the second most commonly used prosodic feature in the NC group and the least-used one in both the MCI and AD groups.

#### 4.2.2. Non-verbal acts

A total of 34 types of non-verbal acts were identified and categorized into head movement, hand movement, body movement, or facial expression. Next, the study calculated the average frequency of each category in a single RIF performance. The results reveal that the three primarily used non-verbal acts in the NC group were head-shaking, head-scratching, and body leaning away; head-shaking, frowning, and looking at the examiner in the MCI group; and head-shaking, looking at the examiner, and head-turning in the AD group. Hence, head-shaking was the most common non-verbal act used by all three groups when performing the RIF.

As for the average number of non-verbal acts used by older adults in each RIF, the MCI group ranked first with 2.36 non-verbal acts, followed by the NC group with 1.75. The AD group, with 1.46 acts, ranked least. The Kruskal-Wallis test found no significant difference in the number of non-verbal acts when the RIF was performed among the three groups (*p* = 0.068, >0.05).

### 4.3. Elements affecting the performance of the RIF

According to the basic tenet of Huang’s (2022) simulative modeling outcome and working scheme for multimodal pragmatic analysis of live illocutionary force, intentional states, and primary emotions are discussed in detail to address the possible affecting elements in older adults’ performing RIF in this section.

#### 4.3.1. Intentional states

**Figure 4:**
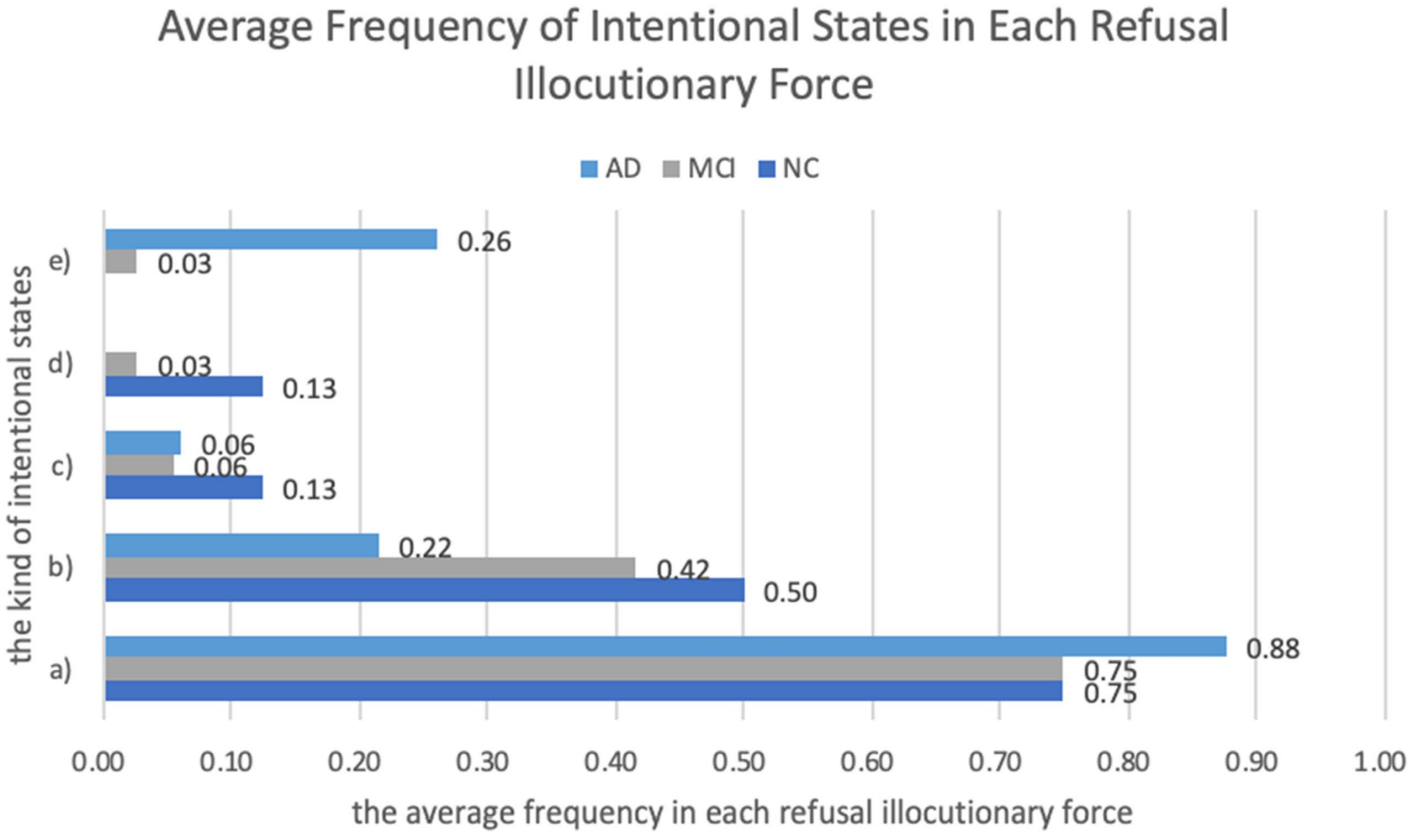
Average frequency of different types of intentional states in each RIF.

Based on the multimodal cues of speech content, prosodic features, and non-verbal acts, there were altogether five types of intentional states identified in the data: (a) the intention of showing one’s inability or difficulty to carry out or continue the required task, (b) the intention of maintaining one’s face, (c) the intention of earning some thinking time, (d) the intention of asking for more information, and (e) the intention of getting the examiner to stop asking. When performing the RIF, older adults may have one intention alone or multiple intentions at the same time. The average frequency of each type of intentional state in a single RIF is shown in Figure 4.

As Figure 4 depicts, two of the five intentions were quite common to the NC, MCI, and AD groups, namely (a) the intention of showing one’s inability or difficulty to carry out or continue the required task and (b) the intention of maintaining one’s face. In all three groups, these two intentions often appeared in combination: when performing the refusal speech act, older adults usually made one utterance to show they were unable or felt difficult to participate in the action proposed by the interlocutor, and added some expressions to make the refusal behavior acceptable and thus maintain their face. Besides, the intention of (c) earning more time to think and (d) asking for more information were more frequently found in the NC group than in the other two groups. The MCI and AD groups also had another intention: (e) getting the examiner to stop asking and then terminating the assessment.

#### 4.3.2. Primary emotion

The four common primary emotions in our data were “default,” “sorrow,” “worry,” and “disgust.” The “sorrow” emotion was produced when older adults felt disheartened or embarrassed about their inability or difficulty to carry out the task or to provide a reasonable answer. The emotion of “worry” often occurred when older adults felt anxious or worried about their memory as well as other cognitive abilities. The “disgust” emotion was produced when older adults were unwilling to participate in the action proposed by the examiner or even resisted the whole cognitive assessment process. As for the emotion of “default,” i.e., no obvious emotional cues were found, it appeared in our data since the regular question-answer sequence, such as Yes/No question and answer, did not produce apparent emotions.

The most common primary emotion among all three groups when performing the RIF was “default.” Nonetheless, the percentage of “default” emotion among all emotions varied in the three groups, with 37.5% in the NC group, 51.4% in the MCI group, and 69.2% in the AD group. When it comes to the second common primary emotion, the NC and MCI groups usually produced the emotion of “sorrow,” while the AD group showed the emotion of “disgust” more often.

## 5. Discussion

### 5.1. Possible reasons behind the frequency distribution of different levels of RIF

Studies found that individuals with cognitive impairment (usually at the early stage) may use some communicative coping behaviors to display a positive self-identity or to save face, therefore avoiding being framed as deficient or socially unacceptable when having difficulty carrying out cognitive tasks (Saunders et al., 2011). Excuse-making is one of the common communicative coping behaviors used by patients with cognitive impairment. By making excuses, individuals shift the causal attribution of adverse outcomes from the individual ability to other relatively less important sources (Snyder and Higgins, 1988). Performing Level I (the speech content shows refusal but is not relevant to any subtask or the whole assessment) and Level II (the speech content shows the reluctance to perform the task with a task-related reason or excuse) of the RIF is implicitly displaying the refusal behavior by making relevant or irrelevant excuses. Therefore, it can be inferred that the RIF in the MCI group resorted to face-saving pragmatic strategies such as excuse-making to avoid being perceived as cognitively incompetent and to maintain their positive self-identity when performing the RIF, and hence in our study, the MCI group performed Level I and Level II of the RIF significantly more frequently than the NC group.

However, cognitive decline at different stages might lead patients to perform the refusal behavior with different manifestations in the assessment. For instance, severe cognitive deficit may result in a complete non-response to certain items in the assessment, while low-level refusals (Level I and Level II) that might manifest as excuse-making, etc., are also unattainable. This may explain both why the AD group performed fewer low-level (Level I and II) RIFs than the other two groups, and why the AD group performed more high-level (Level III and IV) RIFs compared to low-level refusal. Besides, the absence of Level I in the AD group may be related to the impaired pragmatic ability of AD patients, since making irrelevant excuses to refuse requires more advanced pragmatic strategies than making relevant excuses. The cognitive and language impairment of AD patients can also explain that: (1) though RIF at Level III was performed by all groups, the AD group had the highest frequency and (2) only the AD group performed Level IV of the RIF.

Given the frequency of gestures at all levels by the different groups, the output is also relevant to older adults’ cognitive status. As we know, in light of the ontogeny and phylogeny of communication, gestures precede verbal speech. For instance, before speaking their first words, infants’ gestures in communication are a window into their preverbal cognitive and social skills development (Vallotton, 2011). Many scholars argue that it is possible that gestures remain a primitive means of communication. In other words, individuals’ language development is intricately entangled with cognitive maturation. When considering that the AD group had the lowest number of gestures (1.46) and the MCI group had the higher number (2.36), we would like to propose that the influence of pragmatic ability on the use of gesture may be at least more indirect than that of speech content. One step further, the use of gesture would be later negatively influenced, which is after all the reflection of progressive cognitive change, and it follows the change of verbal production. Interestingly, this might be the “mirroring sequence” in contrast to the previously mentioned individuals’ communicative ability in which gestures precede verbal speech. This question is out of this paper’s scope, but it is worthwhile being further explored with more empirical studies.

### 5.2. Multimodal features of performing RIF

#### 5.2.1. Prosodic features

Prosody is one of the most commonly studied markers in the research of patients with neurodegenerative diseases (Martínez-Nicolás et al., 2021). Features such as pauses, accents, and speech rates have been used to distinguish between clinically defined groups (Gabani et al., 2009). This study found that both the NC group and the MCI group used stress most often, while the AD group preferred to use no prosodic features when performing the RIF (Figure 3). The differences indicate that the NC and MCI groups were possibly close in their ability to use stress to convey emotional meaning, while this ability was significantly degraded in the AD group compared to the other two groups.

Excerpt 1 (NC patient) and Excerpt 2 (MCI patient) are examples of older adults using stress when performing the RIF. The stressed words are underlined. In Line 2, Excerpt 1, the word “好” (“many”) is stressed to indicate that she knows numerous kinds of fruit and she just fails to think of them at the moment, which is a behavior of saving face and masking her inability to continue the task. In Excerpt 2, the stressed word “什” (“anything”) emphasizes the MCI patient’s unwillingness to participate in the task and also strengthens the effect of refusing.

Excerpt 1

1 Examiner (E): 还有吗?

‘Are there any more?’

2 Patient (P): 有啊(低头)，好多，想不起来了(捂脸;笑声)。

‘Yes <lowering head>, there are many more, but I cannot think of them. <covering face; laughter>.’

Excerpt 2

1 E: 想一下我刚刚说了哪些?

‘What words did I just say?’

2 P: (笑声)我什么都忘了嘛(身体仰离)。

‘<laughter> I have forgotten anything <covering face; leaning away>.’

3 E: 想想看，想想看，没事，不着急。

‘Think about it, it’s fine, no hurry.’

Laughter is fundamentally an interactional mechanism and has the potential to convey various communicative meanings (Kovarsky et al., 2009; Pressman et al., 2017). Laughter also shares diverse conversational functions such as showing an understanding or stance on what the interlocutor is saying, inviting reciprocal laughter, displaying a willingness to cooperate to end the conversation topic, mitigating embarrassment and potential face-threat, and so on (Jefferson, 1979; Lindholm, 2008; Holt, 2010; Ticca, 2013; Dionigi and Canestrari, 2018). However, some laughter functions differently from the others. According to Holt (2010), when appearing around a potentially termination-related sequence, laughter can function as an invitation to the interlocutor to cooperate in topic termination, which is usually realized by shared laughter. It is also true in our data. In Excerpt 3, an AD patient is performing the calculation task. What happens in Line 4 is the patient’s second refusal speech act (the first is in Line 2). In Line 4, the patient’s utterance seems to be actively cooperating with the examiner, but in fact is merely repeating what the examiner has said in Line 1 without producing more useful information regarding answering. More importantly, laughter is used right after the utterance, which can be seen as an eagerness to close the current topic.

Excerpt 3

1 E: ……我不会给您找零钱啊，您需要付给我13元整。

‘……I will not give you any change, so you need to pay me exactly 13 yuan.’

2 P: 这个我不知道。

‘I do not know about this.’

3 E: 嗯，你说说看啊，你怎么付给我啊。

‘Well, tell me how you are going to pay me.’

4 P: 那我这是付-那多少钱付是-你要不给钱，那我就给你正好的钱呗 **(笑声)**。

‘So I’m paying… how much is that… If you do not pay, I’ll pay you exactly what you need <**laughter>**.’

5 E: 那您告诉我怎么给啊。

‘Then tell me how you will pay.’

#### 5.2.2. Non-verbal acts

Non-verbal acts, as a multimodal language resource, and speech are intimately connected in human interaction. Non-verbal acts enable people to engage in meaningful interactions when they cannot communicate by talking (e.g., waving farewell behind a closed window); they frequently occur as an accompaniment to speech in face-to-face communication (e.g., nodding in agreement) (Brown and Prieto, 2017).

The three groups used different types of non-verbal acts. According to the three most-used non-verbal acts summarized in Section 4.2, the most commonly used non-verbal acts of the MCI (head-shaking, frowning, and looking at the examiner) and AD (head-shaking, looking at the examiner, and head-turning) groups belong to the categories of head movement and facial expression. In contrast, the NC group (head-shaking, head-scratching, and body leaning away) applies non-verbal acts involving categories of head, hand, and body movements, reflecting the fact that the NC group can mobilize a relatively greater variety of non-verbal acts and a wider range of movements.

The frequency of non-verbal acts also varied among the three groups. The study found that the MCI group used non-verbal acts most frequently when performing the RIF, followed by the NC group and finally the AD group. According to Perkins (2007), individuals constantly choose, intrapersonally or interpersonally, what and how much to signal with both verbal and non-verbal resources such as intonation and non-verbal acts in spoken communication. The language abilities of patients with cognitive impairment are also reduced. When experiencing language impairment, the patient’s brain will activate pragmatic compensation mechanisms, using the sensory-motor system to compensate for deficiencies in the language system, which is manifested externally in the phenomenon of patients substituting non-verbal acts for verbal expressions. This kind of pragmatic compensation mechanism provides a plausible explanation for the finding that the MCI group, on average, used the highest number of non-verbal acts in each RIF. For example, in Excerpt 4, an MCI patient is performing the abstraction task and should answer the question about which category a train and a boat belong to. The patient refuses to give an answer by showing her lack of understanding of the “category.” The patient alternatively uses non-verbal acts such as reaching out, shaking head, and frowning to help with the meaning expression. The hand movement of reaching out appears three times with different compensation referees by activating her sensory-motor system to compensate for her deficit in the verbal system. Moreover, the patient also frowns and shakes her head to make her refusal behavior more obvious and intense to the examiner. In this case, the pragmatic compensation mechanism takes place at the intrapersonal level.

Excerpt 4

1 E: 您再换一种方法说一下它们还属于什么类别。

‘Please tell me another category they belong to.’

2 P: (3 s) 换一种方法还属于什么类别?我可能没-不理解 **(伸手；皱眉；摇头)** 这个东西。这个属于哪个 **(伸手；看向评估员)** 类别-类别的我不，不理解 **(摇头)**， 不懂 **(伸手；摇头)**，就是不懂。

‘(3 s) Another category? I probably did not… do not understand **<reaching out hand; frowning; shaking head>** this. To which category **<reaching out hand; gazing at the examiner**> do they belong… I do not, do not understand **<shaking head>**, I do not get <reaching out hand; shaking head>, just do not get it.’

3 E: 哦好。

‘OK.’

However, the interpersonal level of the mechanism is more likely to be activated in the AD group, as what happened in Excerpt 5, since AD patients may find themselves in a state of intrapersonal disequilibrium, which is manifested by the inability of AD patients to mobilize the semiotic and sensorimotor systems for pragmatic compensation, thus leading to a reduction of non-verbal acts when performing the RIF. Therefore, AD patients may use fewer non-verbal acts than MCI patients and cognitively healthy older adults, in other words, activating interpersonal compensation more.

Excerpt 5

1 E: 请您说说香蕉和橘子属于什么类别。

‘Please tell me which category a banana and an orange belong to.’

2 P: 这个不知道。这个不知道。

‘I do not know about this. I do not know about this.’

3 E: 你先想一想啊，就这两个他们属于哪一类的?

‘Think about it for a moment, which category do they belong to?’

4 P: 香蕉是凉性的吧，对不对?

‘Banana is cool fruit, right?’

Excerpt 5 is an AD patient doing the abstraction task. When refusing to offer a reasonable answer to the examiner’s question in Line 1, the patient just hedges by saying that she does not know verbally. Compared with the MCI patient in Expert 4, the AD patient uses no non-verbal act to compensate for the vague pronoun “this” in Line 2. Besides, the main interpersonal compensation is achieved by the examiner who encourages the patient to produce an appropriate answer to move the conversation along, as can be seen in Line 3.

Since there was no significant difference among the three groups in the number of non-verbal acts when performing a RIF, pragmatic compensation may play a limited role in adjusting non-verbal acts in spoken communication. However, it remains undeniable that non-verbal acts play very different roles in the expression of speech acts by different people, and the contribution of these multimodal resources in assisting speakers in spoken communication is variable with multiple possibilities (Huang, 2022), largely due to individual differences and social and cultural influences.

### 5.3. Elements affecting the performance of RIF

#### 5.3.1. Intentional states

It is found that, in all groups, the intention of showing one’s inability or difficulty to carry out or continue the required task and the intention of maintaining one’s face often appeared in combination. For instance, in Excerpt 6, the MCI patient refuses the examiner by saying she is unable to figure out the answer at the very beginning. She then reasons that none of her family members has gone to school. As a school calendar may remind one of the days of the week, we presume that what she means is that she does not need to take her family members to and from school, so she is not sure what day of the week it is. After a 1.5-s silence, she adds an explanation to her previous statement to enhance the credibility of her reasoning. This explanation mitigates the face-threatening effect of admitting her inability or deficiency and hence maintains her face.

Excerpt 6

1 E: 你想一下今天应该星期几。

‘Think about it, What day of the week is it?’

2 P: 想不出来(转头)，家里也没有人读书的。(1.5 s) 都是大人。

‘I cannot < turning head>. No one in the family goes to school now. (1.5 s) It’s all adults.’

3 E: 那您告诉我，这是什么地方啊?

‘Then tell me, where is this place?’

The intention of showing one’s inability or difficulty to carry out or continue the required task can appear alone, in the utterance of “(我)不知道” (“(I) do not know”), “(我)想不起来/想不到/想不出” (“(I) cannot think of”), “(我)忘了/不记得了” (“(I) forgot/ cannot remember”). For convenience, “I do not know (IDK)” will be used hereafter to represent this range of responses, since all these responses are actually the unimpeachable accounts provided by the speaker to avoid answering, enforce resistance, and escape from direct challenges (Yu and Guo, 2020). It was found that when using the IDK to refuse, the NC group would generally provide an explanation of their refusal, or with a response that is weaker than the expected answer. Although the MCI and AD groups also followed the two types of structures, most of their IDK appeared alone, such as Line 2 of Excerpt 5, or repeated themselves, such as Line 2 of Excerpt 2. According to Garfinkel (1967), when the speaker is unable to provide an answer, simply responding with IDK is not “ethonomethods,” and is contrary to social norms (Yu and Guo, 2020). Therefore, compared with the NC group, the MCI and AD groups’ ability to self-orientate to the conversation structure and the social norms behind it was diminished.

**Figure 5:**
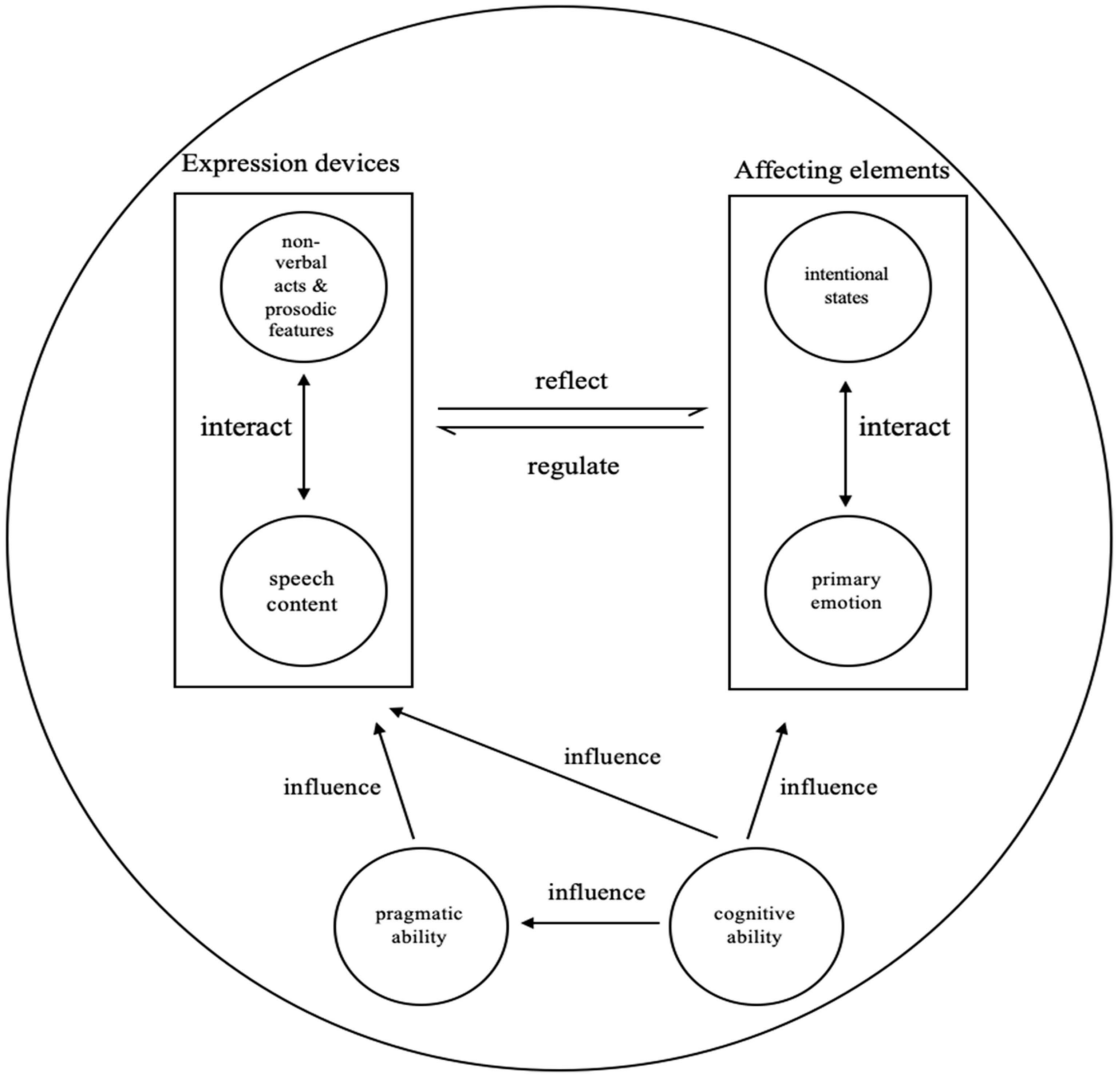
The interactive mechanism of performing the RIF.

Besides, according to Figure 5, the intention of earning more time to think and asking for more information were more frequently found in the NC group, which reflects the fact that the NC group tended to have a relatively low degree of resistance and still showed willingness to cooperate with the task. The AD group had the intention of getting the examiners to stop asking, indicating a relatively high degree of resistance with the purpose of terminating the assessment in this group.

#### 5.3.2. Primary emotion

The study found that the primary emotion of “default” showed up more frequently in the MCI and AD groups than in the NC group, indicating that except for those regular question-answer sequences which were less likely to produce emotion, the MCI and AD groups tended to show no obvious emotion when they were supposed to. This finding may be explained by previous research that has proven that MCI and AD patients are more likely to have reduced emotions and diminished motivations, as well as the neuropsychiatric symptom of apathy (Robert et al., 2009; Pink et al., 2015; Ma, 2020).

In addition, the NC and MCI groups usually produced the emotion of “sorrow,” while the AD group showed the emotion of “disgust” more often. These emotional states reflect that the AD group had a higher level of RIF than the NC and MCI groups. Moreover, the difference in emotions corresponds to the intentional states in our data that the NC and MCI groups sometimes intended to ask for more information or gain more time to think, while the AD group often intended to get the examiner to stop asking.

### 5.4. The interactive mechanism of performing the RIF

During interpersonal conversation, multiple expression devices interact dynamically and synergistically to help older adults perform an illocutionary force. In our data, the speech content, non-verbal acts, and prosodic features on the one hand interact with each other to express the RIF, and, on the other hand, reflect older adults’ intentional states and primary emotion that cannot be observed from the outside. The intentional states and primary emotion, in turn, regulate older adults’ use of expression devices including speech content, non-verbal acts, and prosodic features. Besides, older adults’ cognitive ability and pragmatic ability also influence their use of expression devices. The interactive mechanism is displayed in Figure 5.

We selected two cases (Excerpts 1 and 7) regarding different cognitive ability and different tasks, to display how older adults mobilize various expression devices when performing a RIF. Excerpt 1 is an NC older adult carrying out the fluency task.^4^ The older adult has named several fruits before, and the examiner is encouraging her to name more by asking if she remembers. The older adult’s answer in Line 2 shows that she is performing Level III of the RIF, since the older adult says that she cannot think of any more fruits. When performing the RIF, the NC older adult uses non-verbal acts and prosodic features to help express the speech content and to make her intentions more obvious to the examiner. When asked if there are any other fruit, the older adult does not actually think of fruit that has not been mentioned yet. However, intending to maintain her face and her identity of being cognitively competent, she answers with “有啊, 好多” (“Yes, there are many more”) to show her wide knowledge about the fruit. At the prosodic level, the stress is put on the word “好” (“many”) to strengthen her wealth of knowledge. After maintaining her face, she discloses to the examiner that she is unable to think of any fruit, and hence performs the RIF. Both non-verbal acts of lowering the head and covering the face display the older adult’s resistance to continue the task, as well as her embarrassment at exposing her inability to carry out the task as the examiner expects. Lastly, the older adult ends the utterance with laughter, to mitigate her embarrassment and to invite the examiner to close the current task together.

Excerpt 7

1 E: 您就想想看，我刚才让您怎么做的啊。

‘Think about what I just asked you to do.’

2 P: 哦::(转头；放下笔)搞不清。

‘Oh::<turning head; putting down the pen> (I) cannot figure it out.’

3 E: 啊好没关系啊，接下来…

‘It’s okay, next…’

Excerpt 7 shows an AD older adult performing the executive function task.^5^ After the instructions, the patient performs the RIF by putting down the pen and showing her inability to execute. This is a RIF of Level III. The older adult first displays her resistance with the stressed and extended “哦::” (“Oh::”), along with the movement of turning her head, making the older adult’s intention of getting the examiner to end the task clear and powerful. Besides, the movement of putting down the pen shows the older adult’s resistance and unwillingness much more obviously, since the task cannot be carried out without a pen. The older adult then performs the RIF with the short utterance “搞不清” (“(I) cannot figure it out”). The separate “(I) do not know” sequence without any explanation or weaker answer followed violates the social norm in interpersonal communication, which demonstrates the AD older adult’s degraded pragmatic ability.

## 6. Conclusion

This study examines how 109 examples of refusal speech illocutionary force were performed by nine Chinese older adults with different cognitive abilities in the cognitive assessment with a multimodal approach, including the features, expression devices, and influencing factors of different degrees of RIF, to create an initial exploration of the patterns and mechanisms of refusal behavior of Chinese older adults in the specific clinical context of the cognitive assessment. There are several thought-provoking findings that are open to discussion: (1) When categorizing the RIF into four levels, Level III of the RIF, of which the speech content reflects an inability or difficulty to perform or continue the task, was most frequently used by older adults of all cognitive abilities; both the degree and the frequency of the RIF were highest in AD older adults, followed by MCI older adults, and lowest in cognitively healthy old adults. (2) MCI older adults used non-verbal acts most frequently to compensate for the impaired verbal system when performing the RIF, followed by cognitively healthy older adults, and AD older adults used non-verbal acts less often due to the degraded pragmatic compensation mechanism; the frequency of using prosodic features was also related to the speaker’s cognitive ability. (3) The older adults’ intentional states and primary emotion resonated with the degree of refusal behavior, especially with AD older adults tending to have the intention of getting the examiner to terminate the conversation as well as the emotion of resistance. (4) Regardless of cognitive ability, multiple expression devices interacted dynamically and synergistically to help the speaker perform each RIF. The older adults’ non-verbal acts and prosodic features interacted with each other to express the RIF, and to reflect the older adults’ intentional state and emotion too.

There are some limitations in this study. Firstly, this is pioneering research with a small-scale research sample. A larger sample will help confirm or further explore these findings, making the patterns of Chinese older adults’ refusal behavior and the underlying mechanisms clearer. Secondly, this is a study with regional features, most of whose participants are Shanghai locals. The way refusal behaviors are performed may be influenced by social and cultural elements. Therefore, the findings may not necessarily represent the entire Chinese senior population. Lastly, the current study focuses on the illocutionary act of refusal, not considering too much the examiner’s recognition or reaction toward the refusal behavior.

Despite these limitations, the present research should be considered as a preliminary study that explores the external patterns and internal mechanisms of Chinese older adults’ refusal speech act in the cognitive assessment from the multimodal pragmatic perspective, which can provide inspiring implications for scholars interested in older adults’ refusal speech act, and for the cognitive assessment examiners who need to provide timely care to the older adults, to promote the efficiency of the cognitive assessment. Based on the present research, future studies could be carried out from various perspectives. For example, further analysis is needed to investigate the distinguishing features or patterns of the four levels of RIF established in this study. Another topic for future research is how to analyze the refusal behavior in combination with other pragmatic research issues such as face-saving, especially in certain contexts. Besides, future research could pay more attention to the examiner’s action after the patient’s refusal behavior, which is vital for the completeness of the perlocutionary act.

## Data availability statement

The original contributions presented in the study are included in the article/supplementary material, further inquiries can be directed to the corresponding author.

## Ethics statement

The studies involving human participants were reviewed and approved by Research Ethics Committee of School of Foreign Languages, Tongji University [No. tjsflrec202101]. The patients/participants provided their written informed consent to participate in this study. Written informed consent was obtained from the individual(s) for the publication of any potentially identifiable images or data included in this article.

## Author contributions

LH designed the whole study and wrote the draft of the paper. HQ conducted the statistics and wrote the draft. DZ designed the study, recorded the data, and wrote the draft. All authors contributed to the article and approved the submitted version.

## Funding

This study was funded by the Key Project of National Language Committee of China “A Machine-learning-based Study of Linguistic Markers of Older Adults’ Cognitive Impairments”, and the Major Project of the National Social Science Foundation of China (NSSFC) “The Study of Norm, Evaluation and Intervention of Chinese Elders’ Linguistic Competence” (No. 21&ZD294).

## Conflict of interest

The authors declare that the research was conducted in the absence of any commercial or financial relationships that could be construed as a potential conflict of interest.

## Publisher’s note

All claims expressed in this article are solely those of the authors and do not necessarily represent those of their affiliated organizations, or those of the publisher, the editors and the reviewers. Any product that may be evaluated in this article, or claim that may be made by its manufacturer, is not guaranteed or endorsed by the publisher.

## Footnotes

^1^https://www.mocatest.org

^2^The "expert" in this study refers to specialized researchers who have been educated and trained in linguistics, especially in the field of pragmatics, discourse analysis, or multimodal linguistics. Linguistic experts familiar with these fields of research can quickly understand the substance, purpose, and annotation scheme of this study, and can make targeted suggestions for annotation revision.

^3^A third expert was invited to discuss the controversial results with the two experts together, after which the level of each RIF was determined.

^4^In the fluency task, the older adults are asked to name as many fruits as they can think of within one minute. Points are allocated according to the number of fruits the older adults name. Repeated words are not scored.

^5^The executive function task requires the older adult to draw a line alternating between a square with a number and a square with dots in increasing order. The line should begin at the square with the number 1 and end at the square with six dots.
